# Somatosensory Electrical Stimulation Does Not Improve Motor Coordination in Patients with Unilateral Knee Osteoarthritis

**DOI:** 10.3390/jcm8020259

**Published:** 2019-02-19

**Authors:** Menno P. Veldman, Julia F. Item-Glatthorn, Rosa M.S. Visscher, Tibor Hortobágyi, Nicola A. Maffiuletti

**Affiliations:** 1 Center for Human Movement Sciences, University Medical Center Groningen, University of Groningen, 9713AV Groningen, The Netherlands; menno.veldman@kuleuven.be (M.P.V.); t.hortobagyi@umcg.nl (T.H.); 2 Movement Control and Neuroplasticity Research Group, Department of Movement Sciences, KU Leuven, 3001 Leuven, Belgium; 3 Human Performance Lab, Schulthess Clinic, 8008 Zurich, Switzerland; julia.item@kws.ch (J.F.I.-G.); rosa.visscher@hest.ethz.ch (R.M.S.V.)

**Keywords:** electrical stimulation, knee osteoarthritis, motor control

## Abstract

Non-surgical treatment of knee osteoarthritis (KOA) is often focused on the motor component of KOA even though there is evidence that sensory dysfunctions play an important role in the impaired control of the affected joint. Excitation of sensory afferents can increase motor function by exploiting the nervous system’s ability to adapt to changing environments (i.e., neuronal plasticity). Therefore, the aim of this study was to explore the acute effects of a single session (30 min) of sensory intervention targeting neuronal plasticity using low-frequency (10 Hz) somatosensory electrical stimulation (SES) of the femoral nerve. We evaluated the effects of SES on the position and force control of the affected knee and self-reported pain in KOA patients (*n* = 14) in a sham-controlled randomized trial. The results showed that SES did not improve measures of lower-limb motor coordination compared to sham stimulation in KOA patients, nor did it improve self-reported knee function and pain (all *p* > 0.05). In conclusion, despite sensory involvement in KOA, the sensory intervention used in the present explorative study did not relieve self-reported pain, which may underlie the absence of an effect on measures of motor coordination. In sum, the present explorative study showed that SES alone does not improve motor coordination in KOA patients.

## 1. Introduction

Knee osteoarthritis (KOA) is a chronic joint disease affecting 10 and 18% of men and women (WHO). KOA causes pain, movement limitations, stiffness, crepitus, and inflammation [1]. Preventative, non-surgical, and non-pharmaceutical treatment of KOA-related pain, movement restriction, and muscle weakness most often involves diet and exercise [2]. Besides the motor component, sensory dysfunctions also impairs the control of the affected joint [3,4].

Sensory information associated with motor behavior originates from either environmental inputs (afference) or an organisms’ own motor actions (reafference). Since the same sensory structures are involved in the processing of both types of sensory information, there is a continuous performer–environment interaction that is required for accurate motor behavior. The component (performer or environment) that is considered to be driving this interaction varies between theoretical frameworks. That is, while ecological psychology’s views on motor control consider information from the environment key for motor behavior, the representation theory assumes behavior originates from the central nervous system and is shaped via an internal model (see [5] for an example and elegant discussion). In this study, we do not aim to provide evidence for one particular theoretical framework but rather capitalize on increasing the effectiveness of the sensorimotor system, specifically focusing on its functionality. That is, we aim to increase sensory function, and in particular, the processing of proprioceptive information that is impaired but often underappreciated [3] in KOA patients, despite evidence that KOA-related sensory dysfunction is a major contributor to motor impairments including, but not limited to, locomotor activities [6].

In the past decades, there has been increasing interest in counteracting such sensory dysfunctions via electrical stimulation interventions in the form of low-frequency and -intensity somatosensory electrical stimulation (SES), which have been shown to relieve pain in KOA patients [7]. Moreover, similar interventions improved functional motor control in healthy and stroke populations [8,9,10]. There is increasing evidence in upper as well as lower limb muscle groups that excitation of cutaneous and proprioceptive afferents through electrical stimulation can promote proprioception in KOA [11], the excitability of motor cortical cells, as well as motor performance in healthy and neurological populations [8,12]. Such SES-induced effects on motor cortical function and motor behavior are hypothesized to be mediated via activation of sensorimotor neural structures reached by activating sensory receptors causing afferent volleys, ultimately reaching the sensorimotor cortex (for a review, see [8]). Such predictions are reinforced by the observation that electrical stimulation of the knee joint did not improve gait kinetics and kinematics or postural control in previous studies [13,14]. The present study examined for the first time whether targeting the afference and reafference more directly through acute SES of the femoral nerve may improve measures of motor coordination in the lower limbs. We hypothesized that SES would improve position and force control in patients with KOA relative to a sham condition [15].

## 2. Materials and Methods

Fourteen patients (8 men; mean age ± standard deviation: 65 ± 5 years; height: 1.72 ± 0.12 m; body weight: 81 ± 16 kg) scheduled for knee replacement due to unilateral KOA and with no pain or previous surgery on the contralateral side voluntarily attended the laboratory on two occasions. The study was conducted according to the Declaration of Helsinki, registered at clinicaltrials.gov (NCT02854176), and the local ethics committee approved the protocol. Participants were informed about the aim of the study and provided written informed consent prior to data collection.

### 2.1. Experimental Protocol

**Figure 1:**
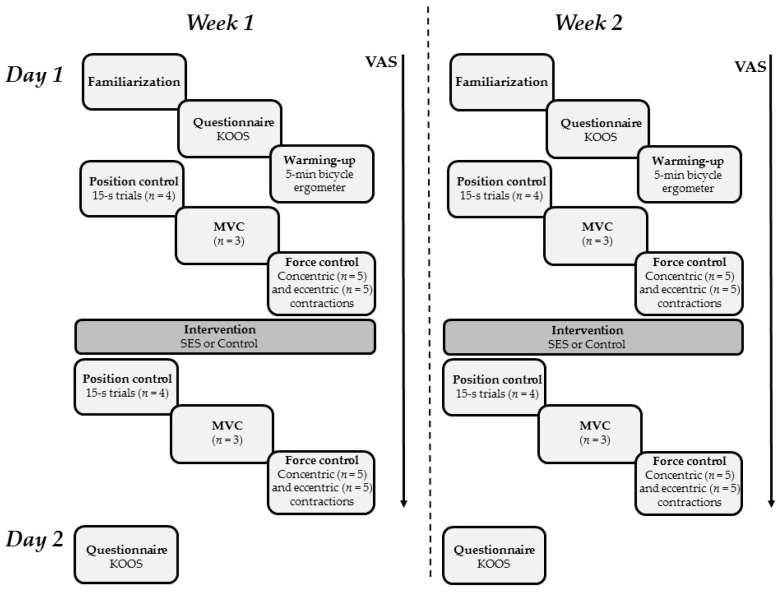
Schematic overview of the experimental protocol. The “*n*” within the boxes refer to the number of trials for that particular measure. KOOS: knee injury and osteoarthritis outcome score; MVC: maximum voluntary contraction; SES: somatosensory electrical stimulation. VAS: visual analogue scale.

The two experimental sessions were identical (except for the type of stimulation), took place 7 ± 1 days apart, and consisted of familiarization with the equipment, a 5-min warm up on a bicycle ergometer, baseline (pre-intervention) measures on the KOA side, the 30-min experimental (SES) or control (sham) intervention, and post-intervention measures. Before and 24 h after each intervention, participants reported daily function and symptoms through the knee injury and osteoarthritis outcome score (KOOS) questionnaire (Figure 1).

### 2.2. Position Control

Lower limb position control was evaluated with the foot positioned on a pedal in a multi-joint closed-kinetic chain exercise model (Allegro, Dynamic Devices AG, Zurich, Switzerland). Patients tracked custom-made sine-wave templates displayed and progressing at 1.9 cm/s on a monitor placed 0.9 m in front of them as accurately as possible via lower limb extension (cursor upward) and flexion (cursor downward) movements. The resistance load was set at 50% of body weight or as high as possible without causing discomfort or pain (mean: 400 N). Patients performed one 15-s-long familiarization trial and four 15-s-long test trials interspersed by 1 min of rest.

### 2.3. Maximal Voluntary Strength and Force Control

Participants were seated on a dynamometer for single-joint open-kinetic chain exercise testing (Biodex System 4 Pro, Biodex Medical Systems, Inc., Shirley, NY, USA) [14]. After warm-up trials, participants performed three isometric knee extensions at 45° of knee flexion with 30 s of rest between contractions. The average of the three contractions was used in the analysis.

**Figure 2:**
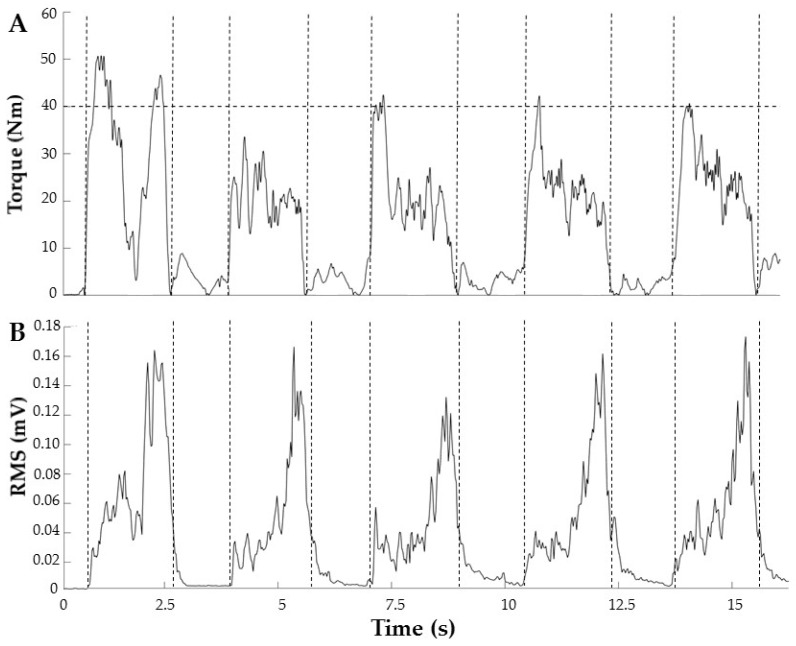
Torque (**A**) and electromyographic RMS (**B**) data from a representative participant during the concentric force control condition. The horizontal dashed line in panel A depicts the target torque and the vertical dashed lines indicate the time windows of subsequent trials. RMS: root mean square.

Thereafter, concentric and eccentric knee extension force control (accuracy and steadiness) were measured at 20°/s between 90° and 15° of knee flexion (trial duration: 3.75 s). The target torque during these trials was calculated so that the relative electromyographic root mean square (RMS) amplitude of the vastus lateralis muscle during position and force matching relative to activity during maximal contractions was comparable to establish comparable effort levels during position and force control measures. The resulting target torque was 35.2 ± 15.3 Nm. Figure 2 shows torque and electromyographic RMS data (concentric force control) from a representative participant. Patients performed five familiarization trials followed by five test trials in each condition with 1 min of rest between conditions. Knee joint torque–position data were digitized and sampled at 2 kHz using a BIOPAC MP100 unit and AcqKnowledge (v.4.1) software (Biopac Systems Inc., Goleta, CA, USA) and stored for offline analysis.

### 2.4. Electromyography Recording

The skin over the vastus lateralis muscle belly was prepared with fine-grained sandpaper and alcohol prior to electrode positioning. Surface electromyographic activity was recorded at 4 kHz during the position and force measures using wireless Trigno parallel bar electrodes (bandwidth: 20–450 Hz; amplification: 909; channel noise < 0.75 µV; common mode rejection ratio > 80dB; Delsys Inc., Natick, MA, USA).

### 2.5. Somatosensory Electrical Stimulation

While seated with hip and knee angles of ≈90°, SES was delivered to the femoral nerve in the inguinal crease for 30 min through percutaneous electrodes (Dermatrode HE-R, American Imex, Irvine, CA, USA). The femoral nerve is a mixed nerve containing sensory afferents and motor efferents innervating the quadriceps muscles. Electrical square-wave pulses were applied using a Digitimer DS7A constant-current stimulator (Welwyn Garden City, U.K.) in 1-s-trains of five pulses at 10 Hz with a 50% duty cycle. These stimulation parameters previously improved motor learning in stroke patients [9]. Pulse width was 1 ms to target sensory fibers [16] and current intensity was individually adjusted to just below the motor threshold (7.8 ± 4.1 mA), determined as the highest intensity without a motor response in the quadriceps muscle that caused mild paresthesia without pain. The 30-min sham stimulation and SES setup were identical. Electrical pulses were visualized on a computer monitor. However, in the sham condition, the cable was unplugged from the stimulator (invisible to the participant).

### 2.6. Data Analysis

Position control and force accuracy were quantified as the vertical mean absolute deviation between the participants’ cursor and the target for each sample in each trial (Mathworks, Natick, MA, USA, version 2015a). Subsequently, mean absolute deviations were computed for each trial and averaged over all trials as an estimate of position control and force accuracy. Absolute and percent changes were used as indications of increased or decreased position control and force accuracy. In addition, force steadiness was quantified as the standard deviation of the mean absolute differences for each trial (average of five trials), as an additional estimate of force control. The electromyographic RMS amplitude was consistently calculated using EMGworks software (Delsys Inc., Natick, MA, USA) and subsequently expressed as mean and maximal RMS for all position control, force control, and maximum voluntary contraction (MVC) trials separately.

### 2.7. Statistical Analysis

Shapiro-Wilk tests revealed that the assumption of normality was violated. Thus, we used Mann–Whitney U tests to check whether groups differed at baseline. We then performed an intervention (SES and sham) by time (pre- and post-intervention) repeated measures ANOVA on ranked (instead of continuous) data and computed effect sizes (ES) as Cohen’s *d*. Spearman’s rank correlation coefficients were used to examine relationships between pairs of variables. The significance level was set at α = 0.05.

## 3. Results

**Figure 3:**
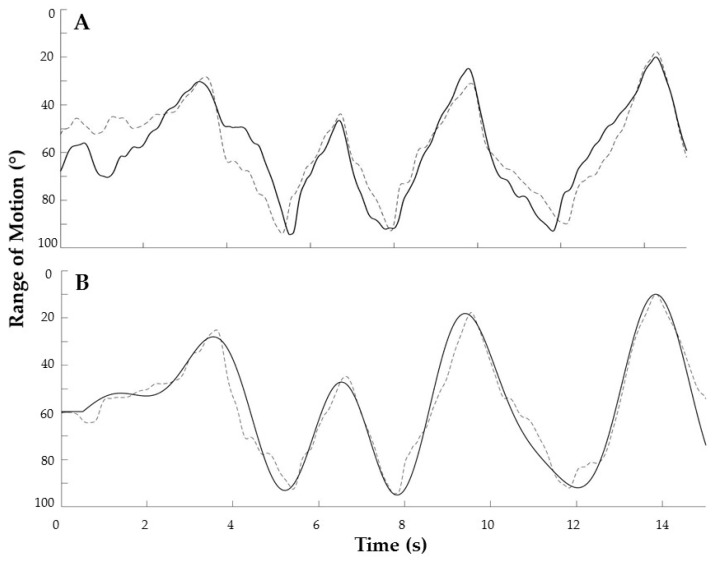
Position control from a representative participant before (**A**) and after (**B**) somatosensory electrical stimulation. The dashed line in panels A and B represents the target position and the thick black line depicts actual patients’ performance.

There were no differences between SES and sham in position control, force control, and maximal voluntary strength at baseline. The ANOVA on ranked scores revealed that SES did not improve position control (7.8% vs. 14.7%, ES: −0.3; see representative results in Figure 3), concentric force control (−14.9% vs. −5.4%, ES: −0.4), eccentric force control (0.4% vs. 10.2%, effect size: −0.4), and maximal voluntary strength relative to sham (3.5% vs. 3.1%, ES: 0.03). There was a time effect for force accuracy (F1,19 = 9.913, *p* = 0.027), but no intervention by time interaction. Next, SES did not modify vastus lateralis electromyographic activity as identified by mean and maximal RMS amplitude. Finally, SES and sham did not differently affect self-reported knee function and pain (KOOS subscores) nor were correlations between outcome measures observed. The ensemble of these data are summarized in Table 1.

## 4. Discussion

The aim of the present study was to examine the acute effects of a single session of SES on motor coordination in patients with unilateral KOA. We discuss the findings of the present study in light of factors that could have contributed to the responses to the SES intervention as observed here and provide considerations for future studies aiming to develop neurorehabilitation protocols for KOA treatment.

In contrast to a series of studies applying high-frequency (100 Hz) stimulation that effectively relieved pain, here we applied a sensory stimulus known to excite proprioceptive and cutaneous afferents (reafference and afference information). Previous studies using SES protocols consisting of 1-Hz trains (internal frequency of 10 Hz, 50% duty cycle), current level at motor threshold intensity in absence of pain and lasting 20–120 minutes improved motor function in healthy adults and stroke patients up to 24% [7,8,9]. However, a similar SES protocol did not improve motor coordination in the present study nor did it increase maximal voluntary strength and self-reported knee function, relieve self-reported pain, or modify electromyographic activity in patients with KOA. These between-study discrepancies could be attributed to differences in the type of skill being investigated. That is, the skill under investigation in previous studies was more fine-grained motor skills in smaller muscle groups and required lower force levels that may be more sensitive to SES. Such predictions are reinforced by the lack of effects of SES on electromyographic activity levels in the present study. However, despite these between-study differences, our data taken as a whole complement data showing limited effectiveness of joint stimulation and high-frequency transcutaneous electrical stimulation for KOA patients [12,15].

While there are differences in the behavioral approaches toward examining SES-induced effects in heterogeneous groups of patients/subjects, differences in the state of the sensorimotor nervous system in these various populations should also be highlighted. It is well-documented that sensory afferents can be affected by KOA-induced joint deformation and inflammation, as well as arthrogenic muscle inhibition [17,18], which could in turn impair proprioception [3]. Because intact sensory inputs are required for skilled behavior (e.g., [19]), it is conceivable that impaired proprioception in KOA patients is a major contributor to motor dysfunctions due to suboptimal integration between sensory and motor information in the primary sensorimotor cortex. Based on extensive anatomical data in rodents and monkeys (for a review, see [8]), we hypothesized that SES would enhance the magnitude of afferent volleys that ultimately reach the sensorimotor cortex. Through these pathways, the sensory and motor terminals involved in force control and motor function indexed by motor coordination and maximal voluntary contraction force in the present study would be selectively activated.

Despite the tenability of this hypothesis and previous evidence for the effectiveness of SES in stroke, dystonia, and healthy participants [9,10,20], the form of SES used in the present study did not improve KOA patients’ motor function, including self-reported function, or relieve pain as compared to sham stimulation (Figure 2 and Figure 3; Table 1). At least part of the reason may lie in the differences in the neurophysiology of sensory and motor systems in KOA in comparison with stroke patients or healthy individuals. First, one may infer from sensory and motor representations in the post- and pre-central gyrus that the afferent input and motor output ratio is less specific in the lower limb of KOA patients compared to this specificity in stroke patients’ upper limb [10]. In addition, while a loss of sensory function contributes to motor dysfunctions after stroke [21], pain is considered the major cause of dysfunction in KOA patients that can be relieved by electrical stimulation [22]. Second, while sensory dysfunction in stroke has a central origin, the origin of sensory dysfunctions in KOA lies in the periphery [3,4]. The present study may indirectly suggest that SES is more effective in increasing sensory function by targeting central structures rather than peripheral structures or receptors.

The question remains in what form, if at all, the SES intervention could be effective in increasing KOA patients’ motor function. Future studies are needed to examine whether multiple sessions of SES have the potential to increase the control of large muscle groups such as the quadriceps. Second, the absence of SES-induced reductions in self-reported pain (see KOOS subscores in Table 1) likely underlies the absence of SES-induced improvements in motor coordination. That is, SES primarily targets supraspinal plasticity and, to a lesser degree, spinal and peripheral neurophysiology [23]. Therefore, we suggest that efficiently combining plasticity-inducing SES with pain-relieving stimulation protocols, such as interferential stimulation [22], could improve motor function in KOA patients. In conclusion, even though the present study may have suffered from a limited sample size given the heterogeneous patient population, SES on its own did not improve lower limb motor coordination in patients with unilateral KOA.

## Figures and Tables

**Table 1:** Motor coordination, maximal voluntary strength, and electromyographic data with self-reported knee function (KOOS subscores).

		SES				Sham			
		Pre-Intervention		Post-Intervention		Pre-Intervention		Post-Intervention	
		Median	IQR	Median	IQR	Median	IQR	Median	IQR
Position control	(°)	7.5	6.7–8.4	6.8	6.5–7.5	7.2	6.2–9.7	6.4	5.5–7.0
	**Mean RMS** (mV)	0.025	0.022–0.058	0.022	0.019–0.029	0.031	0.026–0.049	0.037	0.023–0.080
	**Max RMS** (mV)	0.088	0.054–0.120	0.070	0.046–0.084	0.091	0.067–0.172	0.094	0.066–0.161
Force control concentric	**FA** (Nm)	11.7	9.07–13.17	12.0	9.38–15.11	12.3	7.19–12.79	12.1	9.85–13.84
	**FS** (Nm)	3.7	2.73–4.36	3.4	2.74–4.36	4.3	3.44–6.48	4.3	3.23–5.33
	**Mean RMS** (mV)	0.046	0.030–0.058	0.044	0.023–0.045	0.050	0.035–0.062	0.051	0.034–0.077
	**Max RMS** (mV)	0.104	0.069–0.158	0.093	0.058–0.142	0.122	0.071–0.269	0.118	0.076–0.171
Force control eccentric	**FA** (Nm)	8.7	6.92–10.30	8.4	5.66–10.21	8.9	6.64–10.64	8.4	6.39–9.84
	**FS** (Nm)	5.3	4.02–5.99	5.2	3.60–5.58	4.8	3.75–7.57	4.8	3.84–6.19
	**Mean RMS** (mV)	0.041	0.033–0.045	0.030	0.024–0.039	0.044	0.038–0.061	0.041	0.031–0.052
	**Max RMS** (mV)	0.085	0.057–0.089	0.064	0.047–0.080	0.093	0.089–0.233	0.086	0.064–0.111
Max voluntary strength	(Nm)	87.6	65.6–120.7	106.2	65.8–119.8	86.9	62.9–108.2	84.6	69.3–108.2
	**Mean RMS** (mV)	0.108	0.080–0.144	0.108	0.084–0.143	0.119	0.086–0.171	0.128	0.087–0.158
	**Max RMS** (mV)	0.216	0.139–0.316	0.255	0.157–0.289	0.260	0.169–0.287	0.218	0.170–0.276
KOOS (0 = worst; 100 = best)									
Pain		49	38–58	50	41–61	47	30–63	51	41–62
Symptoms		52	46–54	50	43–60	50	36–60	50	38–55
Activities of daily living		65	50–73	69	53–76	66	61–74	69	47–75
Sport/Recr. activities		25	10–40	30	10–45	20	5–30	30	3–30
Quality of life		34	27–44	38	25–42	31	20–42	28	17–31

FA: force accuracy; FS: force steadiness; IQR: interquartile range; KOOS: knee injury and osteoarthritis outcome score; RMS: root mean square; Recr: recreational.
